# Reversible methotrexate-associated lymphoma of the liver in rheumatoid arthritis: a unique case of primary hepatic lymphoma

**DOI:** 10.1186/s40364-015-0035-2

**Published:** 2015-05-06

**Authors:** Ai Kawahara, Junichi Tsukada, Takahiro Yamaguchi, Takefumi Katsuragi, Takehiro Higashi

**Affiliations:** Hematology, University of Occupational and Environmental Health, 1-1 Iseigaoka, Yahatanishi-ku, Kitakyushu, 807-8556 Japan

**Keywords:** Primary hepatic lymphoma, Methotrexate, Immunodeficiency

## Abstract

Primary hepatic lymphoma (PHL) is an extremely rare disease, frequently associated with viruses such as hepatitis B virus (HBV), hepatitis C virus (HCV), and human immune deficiency virus (HIV). On the other hand, an increased risk of lymphoproliferative disorders (LPD) has been demonstrated in patients treated with immunosuppressive drugs such as methotrexate (MTX) for rheumatoid arthritis (RA). The role of Epstein-Barr virus (EBV) has been discussed in the pathogenesis of the immunodeficiency-associated LPDs. We here describe a RA patient, who developed PHL during RA treatment. The patient was a 64 year-old Japanese male with a 2-year history of RA, who had been treated with MTX at weekly dose of 8–14 mg for 2 years and infliximab (IFX) for 7 months. He presented with a 2 month history of generalized malaise, right hypochondrium pain and fever. Contrast-enhanced computed tomography (CECT) of the abdomen showed multiple irregular and nodular liver masses with a maximum of 13 cm in diameter on the right liver. Biopsy specimens demonstrated CD20-positve diffuse large B-cell lymphoma (DLBCL), but EBV was not identified by EBV-encoded RNA *in situ* hybridization. Serology for HBV, HCV, human T-cell leukemia virus I (HTLV-I), and HIV was negative. His symptoms disappeared following discontinuation of RA treatment including MTX. A drastic regression of the tumor masses was further obtained without cytotoxic chemotherapy. In addition, although the patient had no past history of liver dysfunction before MTX therapy, persistent elevation of liver enzymes has been observed during MTX treatment. These findings show a causative role of MTX in the development of reversible PHL in the patient.

## Background

Lymphoproliferative disorders (LPDs) developed in autoimmune disease patients receiving immunosuppressive therapy are categorized as other iatrogenic immunodeficiency-associated LPD in the WHO classification of tumors of hematopoietic and lymphoid tissues [1]. The relationship between the development of LPDs and immunosuppressive drugs, especially methotrexate (MTX) for rheumatoid arthritis (RA) has been discussed [2,3]. MTX has been the most widely used as an anchor drug for the treatment of RA. The advantages of anti-tumor necrosis factor (TNF) therapy over MTX have been also recognized to reduce RA disease activity. However, it has been shown that the immunosuppressive state induced by these drugs provides a basis for the development of LPDs. Spontaneous regression or disappearance of lymphoproliferative lesions after simple discontinuation of the immunosuppressive drugs further presents the evidence of lymphomagenic potential of the immunosuppressive drugs [3-7].

Immunodeficiency-associated LPDs, possess a wide variety of spectrum with features, but share several clinical characteristics, including frequent involvement of extranodal lesions [5,8,9]. Here, we describe a RA patient treated with low dose MTX, who subsequently developed primary hepatic lymphoma (PHL).

## Case presentation

A 64-year-old man presented with a 2 month history of generalized malaise, right hypochondrium pain and fever. He had a history for appendectomy at the age of 42 and sarcoidosis and RA at the age of 62. He had been treated with MTX at weekly dose of 8–14 mg for 2 years and infliximab (IFX) at 3–6 mg/kg for 7 months for RA. Although there has been no significant history of liver dysfunction before MTX therapy, persistent elevation of liver enzymes has been observed during MTX treatment; aspartate aminotransferase (AST) 36 to 128 U/L (normal value: 13–33) and alanine aminotransferase (ALT) 44 to 93 U/L (normal value: 8–42). Physical examination at the time of admission revealed neither enlarged superficial lymph nodes nor jaundice. The laboratory findings on admission were: white blood cell count 4.3X10^9^/L, hemoglobin 11.1 g/dl, platelet count 147X10^9^/L, AST 92 U/L, ALT 33 U/L, total bilirubin 1.1 mg/dl (normal value: 0.3-1.9), alkaline phosphatase (ALP) 401 U/L (normal range 115–359) and lactate dehydrogenase (LDH) 1,114 U/ml (normal range 119–229). No lymphoma cells were detected in his peripheral blood. A high LDH/AST ratio (12.1) was observed. Soluble IL-2 receptor was 3,465 U/ml (normal range 254–534). Serum carcinoembryonic antigen (CEA) was within normal range, and alpha-fetoprotein (AFP) was slightly elevated (16.3 ng/ml, normal value 10 ng/ml≧). Serological tests for hepatitis B virus (HBV), hepatitis C virus (HCV), human T-cell leukemia virus I (HTLV-I) and human immune deficiency virus (HIV) were all negative.

**Figure 1:**
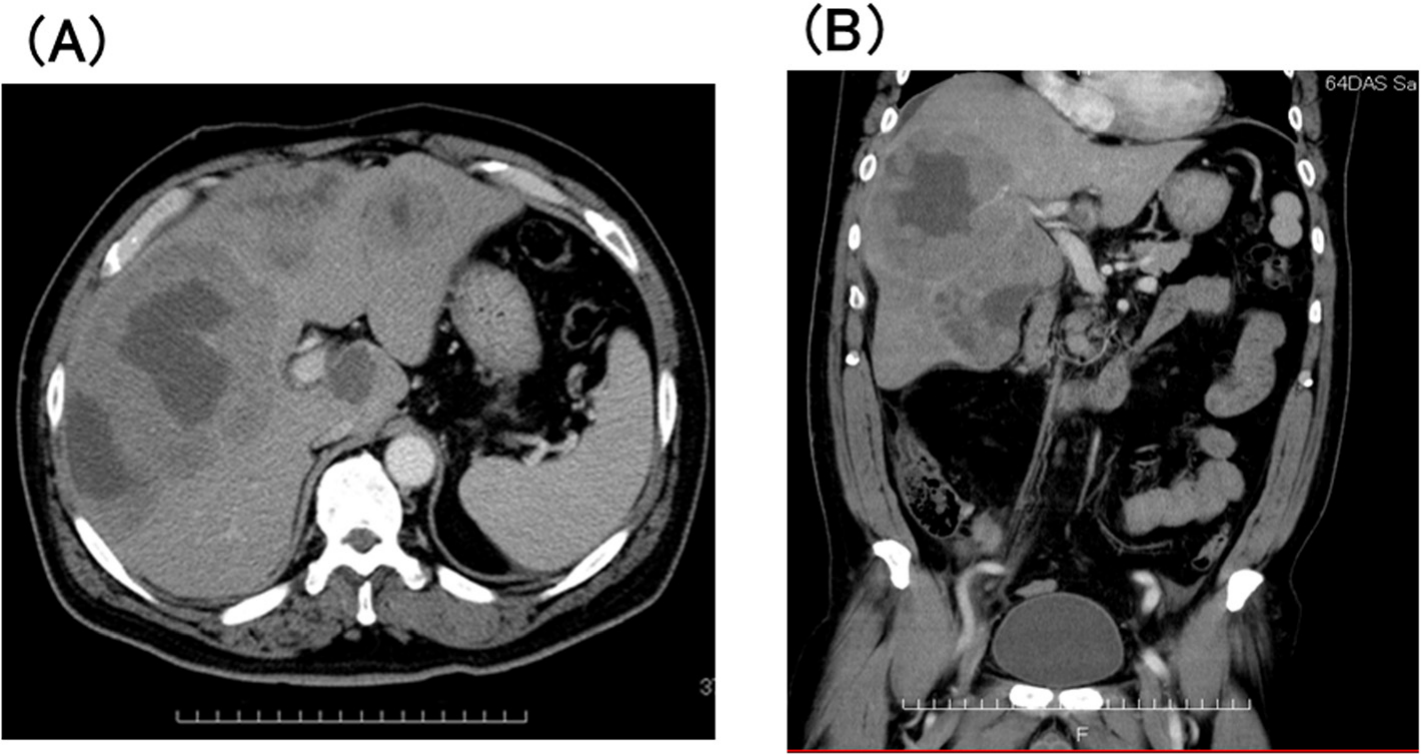
Contrast-enhanced computed tomography (CECT) of the abdomen at the time of admission **(A)** axial view; **(B)** coronal view showed multiple liver masses.

Contrast-enhanced computed tomography (CECT) of the abdomen (Figure 1A and B) revealed multiple irregular and nodular liver masses with a maximum of 13 cm in diameter on the right liver. CECT scan of the brain, neck, chest and pelvis showed no enlarged lymph nodes or masses. Multiple hypoechoic bulky liver masses were observed in ultrasound examination. There was no significant bone marrow infiltration of lymphoma cells.

**Figure 2:**
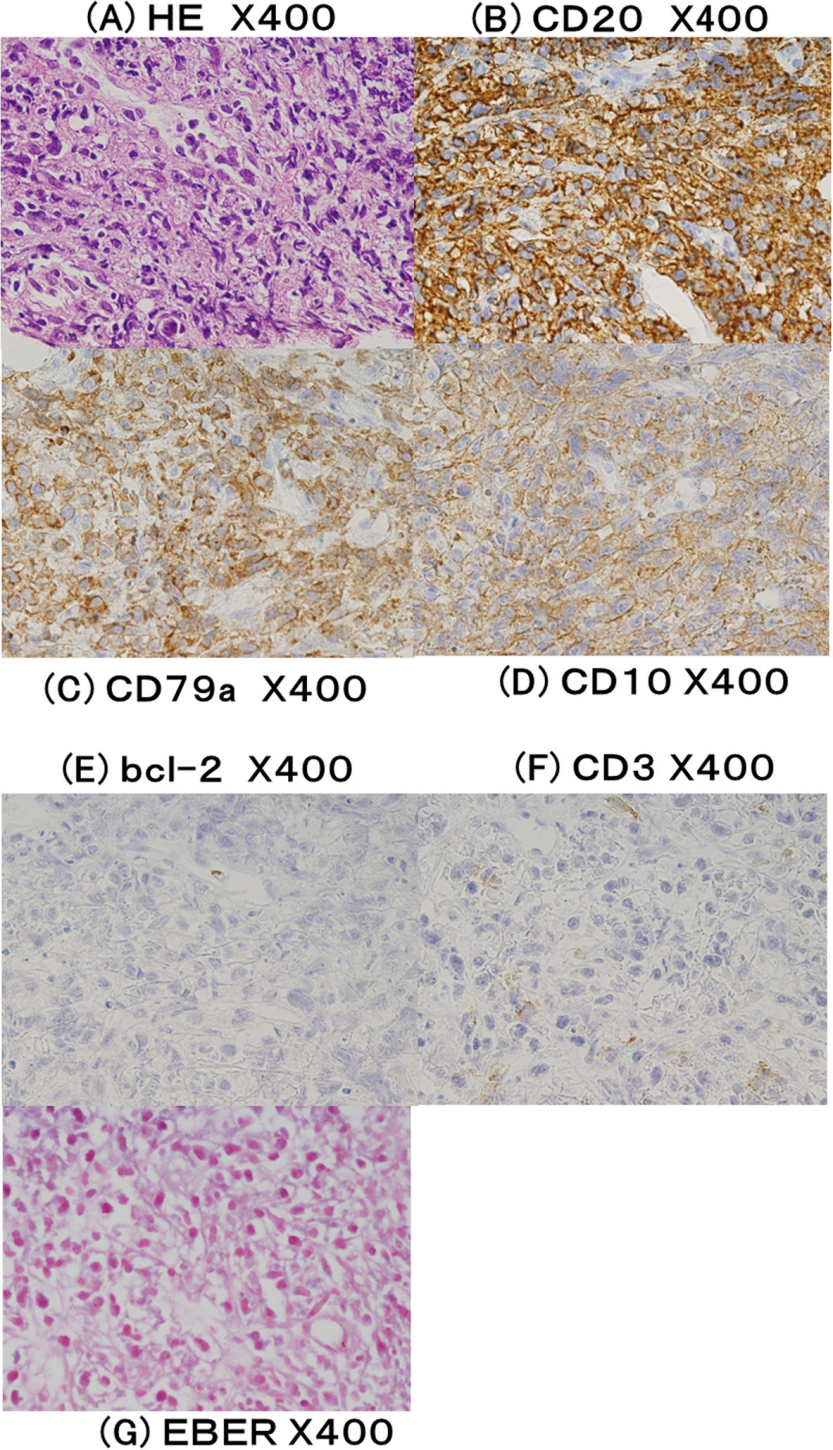
Histopathology of the liver tumor. **(A)** Hematoxylin and eosin (HE) staining, X400; **(B)** anti-CD20 staining, X400; **(C)** anti-CD79a staining, X400; **(D)** anti-CD10 staining, X400; **(E)** anti-bcl-2 staining, X400; **(F)** anti-CD3 staining, X400; **(G)** EBV-encoded small RNAs (EBER) by *in situ* hybridization (ISH), X400. Tumor cells are positive for CD20, CD79a and CD10 and negative for bcl-2, CD3 and EBER.

Diagnosis of PHL was made by using percutaneous tumor biopsy of the liver, which had diffuse infiltration of medium to large lymphoid cells with remarkable nucleoi. The tumor cells were positive for CD20 (Figure 2B), CD79a (Figure 2C) and CD10 (Figure 2D), and negative for bcl-2 (Figure 2E), CD3 (Figure 2 F), and EBV-encoded small RNAs (EBER) by *in situ* hybridization (ISH) (Figure 2G). The findings were consistent with non-Hodgkin lymphoma (NHL), diffuse large B-cell lymphoma (DLBCL), and according to the WHO 2008 classification of lymphoid tissue, he was diagnosed as other iatrogenic immunodeficiency-associated LPDs.

**Figure 3:**
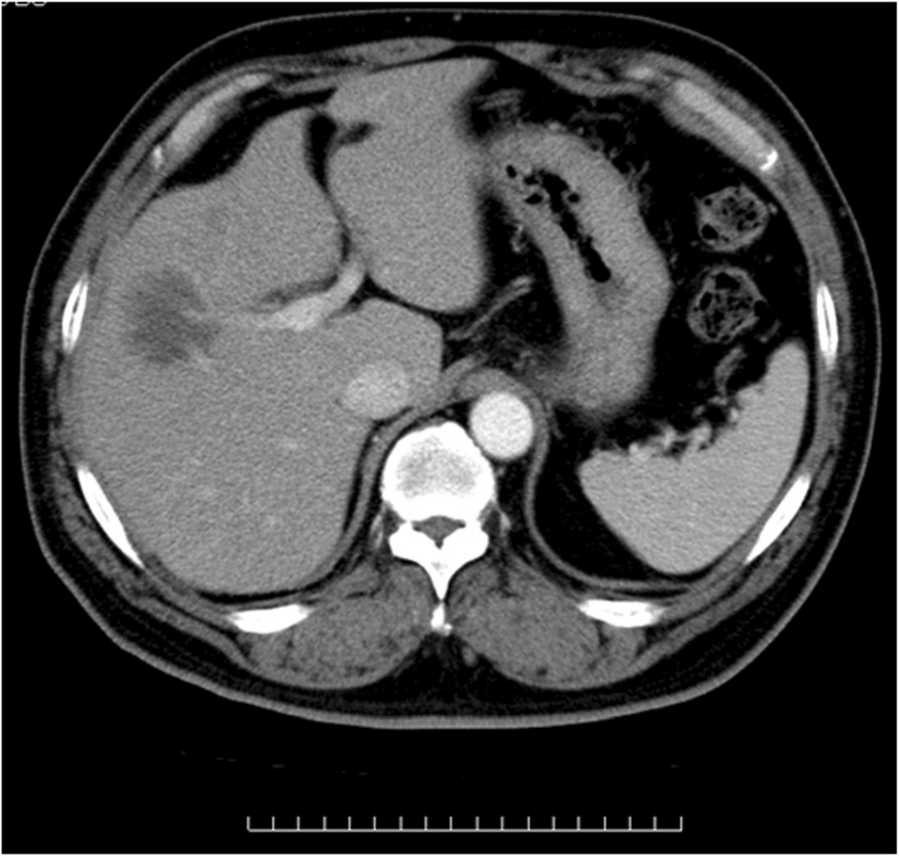
Three months after discontinuation of RA treatment, CECT of the abdomen demonstrated a remarkable regression of the liver masses.

RA treatment including MTX was discontinued. He became asymptomatic, and serum LDH returned to normal levels. His liver function also improved. Oral prednisolone (PSL) was administered at the initial dose of 60 mg/day (0.7 mg/kg) for two weeks and was reduced to 10 mg/day every three days. Three months after discontinuation of RA treatment, the tumor masses dramatically regressed (Figure 3). However, since a small liver mass was still observed, the patient was further treated with a total of 8 cycles of R-CHOP: Rituximab (375 mg/m^2^) with cyclophosphamide (750 mg/m^2^), vincristine (1.4 mg/m^2^), doxorubicin (50 mg/m^2^) and prednisolone (60 mg/m^2^). There has been no sign of recurrence for two years.

## Discussion

PHL is extremely rare, representing 0.4% of extranodal NHL [10] and 0.016-0.06% of NHL [11,12]. The most predominant type of PHL is DLBCL. The other histologic types described in the literature include lymphoblastic lymphoma, Burkitt lymphoma, anaplastic large cell lymphoma, follicular lymphoma and extranodal marginal zone lymphoma of mucosa-associated lymphoid tissue, small lymphocytic lymphoma, mantle cell lymphoma and peripheral T-cell lymphoma [13-15].

Clinical manifestations for PHL were non-specific. Most of patients with PHL had abdominal pain, B-symptoms and hepatomegaly associated with hepatic tumors. PHL patients also have abnormal liver function tests, mostly elevated AST and ALT. A solitary or multiple lesions in the liver was reported [11,14,16].

The etiology of PHL still remains unclear, although viruses such as HBV, HCV, HIV and EBV have been implicated as local factors in lymphomagenesis. A French study of 31 patients with PHL including 22 patients of primary hepatic DLBCL reported that the prevalence of HCV and HBV infection was 21% and 9.5%, respectively [15]. In a Japanese study of 20 patients with PHL, HCV infection was observed in eight of 12 patients of primary hepatic DLBCL (66.7%). The prevalence of HBV infection was 16.7% (2 of 12 primary hepatic DLBCL patients). EBER was positive in two DLBCL PHL patients [17]. Thus, the two studies in France and Japan suggested a potential pathogenic role of HCV infection in PHL development. Persistent or chronic stimulation of the immune system by HCV may result in clonal expansion of B-cells in liver. HCV genome was detected in tumor cells of a patient with PHL [18]. In addition, the development of PHL in HIV-positive patients has been also reported in the setting of immunosuppressive states [19,20].

RA patients have a modestly increased risk of the development of LPDs irrespective of immunosuppressive therapy for the disease. In RA treatment, the most popular immunosuppressive agent is MTX. Since the first report of a RA patient by Ellman *et al.* [2], which describes the development of lymphoma during low dose weekly MTX therapy for 33 months, the development of reversible LPDs has been recognized as a complication of immunosuppression associated with prolonged use of low dose MTX. Spontaneous regression of LPDs following discontinuation of MTX shows the interplay of immunosuppression resulted from MTX therapy with lymphoproliferation. Tumor cells of the reversible LPDs frequently express EBV [3-7].

Approximately 40-50% of MTX-associated LPDs occurred in extranodal sites [5,8,9]. Hoshida *et al.* reported 48 patients with MTX-associated LPDs, of whom the primary site was nodal in 22 patients, extranodal in 23 patients and undetermined in 3 patients [8]. A Study of 37 patients with MTX-associated LPDs reported extranodal organ involvement, including lung, pleura, skin, salivary glands, kidney and pancreas [5].

To our knowledge, four RA patients with MTX-associated PHL have been reported in the English literature [17,21,22]. Among them, one patient reported by Tatsumi *et al*. [21] was negative for HBV, HCV and HIV, and tumor cells of the patient expressed EBER *via* ISH. One patient reported by Miyagawa et al. [22] was negative for HBV, HCV and tumor cell EBER by ISH, but no mention was made of HIV infection status. Moreover, it is interesting to note that in contrast to our patient, the two patients did not show spontaneous tumor regression after the withdrawal of MTX. On the other hand, there was no detailed description regarding the other two patients [17]. Our patient was negative for EBV, HBV, HCV and HIV. MTX sometimes shows a significant hepatotoxicity. Persistent elevated transaminase levels were noted while receiving MTX therapy in our patient. Based upon the findings obtained from the present case, we suggest the possibility that in addition to MTX-induced immunosuppression, chronic liver injury resulted from MTX may play a causative role in PHL development. The fact that both of a drastic regression of the tumor masses and improvement of liver dysfunction were observed following discontinuation of MTX further supports our argument. However, because of the rarity of PHL, further investigation should be required.

## Conclusion

PHL is a rare disease, frequently associated with viruses such as HBV, HCV and HIV. Herein, we present the first case of a reversible PHL occurred during RA treatment. Symptoms of the patient disappeared following discontinuation of RA treatment including MTX. A drastic regression of the tumor masses was further obtained without cytotoxic chemotherapy. The fact that persistent elevations in liver enzymes have been observed during MTX treatment also suggests a causative role of MTX in the development of PHL in the patient.

## Consent

Written informed consent was obtained from the patient for publication of the case report.

## Abbreviations

AFP: Alpha-fetoprotein
ALP: Alkaline phosphatase
ALT: Alanine aminotransferase
AST: Aspartate aminotransferase
CEA: Carcinoembryonic antigen
CECT: Contrast-enhanced computed tomography
DLBCL: Diffuse large B-cell lymphoma
EBER: EBV-encoded small RNAs
EBV: Epstein-Barr virus
HBV: Hepatitis B virus
HCV: Hepatitis C virus
HIV: Human immune deficiency virus
HTLV-I: Human T-cell leukemia virus I
IFX: Infliximab
ISH: *In situ* hybridization
LDH: Lactate dehydrogenase
LPD: Lymphoproliferative disorders
MTX: Methotrexate
NHL: Non-Hodgkin lymphoma
PHL: Primary hepatic lymphoma
PSL: Prednisolone
TNF: Tumor necrosis factor
RA: Rheumatoid arthritis
